# Supplementary material for: Multiple temperatures and melting of a colloidal active crystal
Source: Nat Commun. 2024 Aug 3;15:6574. doi: 10.1038/s41467-024-50937-2 (PMC11297967; doi:10.1038/s41467-024-50937-2)
Supplement: Supplementary file 1 — Supplementary Information [file 41467_2024_50937_MOESM1_ESM.pdf]

Supplementary information of
Multiple temperatures and melting of a colloidal active crystal

Helena Massana-Cid,[1,*] Claudio Maggi,[2,1,†] Nicoletta Gnan,[3,1] Giacomo Frangipane,[1,2] and Roberto Di Leonardo[1,2]

[1]*Dipartimento di Fisica, Sapienza Università di Roma, Piazzale A. Moro 5, 00185 Rome, Italy*
[2]*NANOTEC-CNR, Soft and Living Matter Laboratory, Institute of Nanotechnology, Piazzale A. Moro 5, 00185 Rome, Italy*
[3]*CNR Institute of Complex Systems, Uos Sapienza, Piazzale A. Moro 5, 00185 Rome, Italy*

Supplementary Figures

- **Supplementary Figure 1**

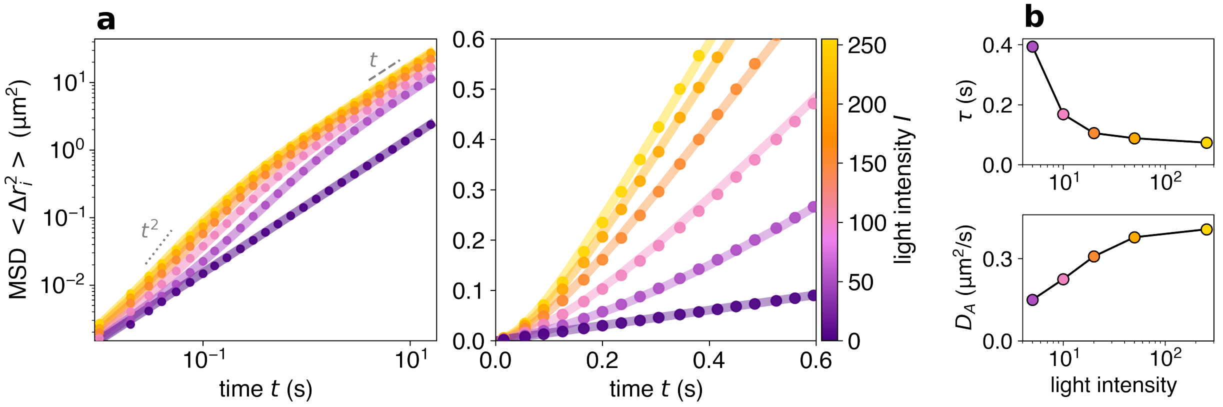

FIG. 1. **Example of calibration of the experimental parameters D_A and τ for one set of experiments.** (a) Mean-squared-displacement in logaritmic and linear scale for different light intensities (see colorbar). Continuous lines correspond to fits to Eq. (11) of the main text. (b) Obtained calibrated fit parameters of the persistence time τ and active diffusion coefficient D_A depending on shined light intensity.

- **Supplementary Figure 2**

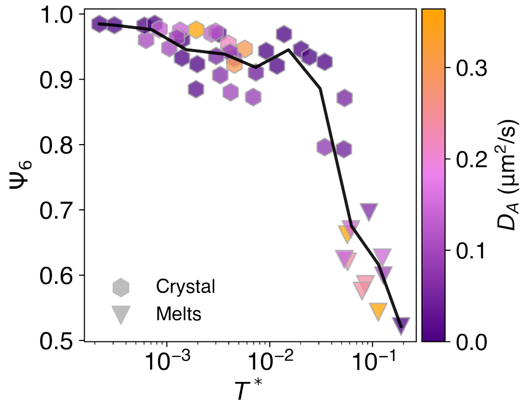

FIG. 2. **Order parameter as a function of the adimensional global temperature.** For all data points of Fig. 3a of the main text, we plot the orientational bond-order-parameter Ψ_6 as a function of adimensional global temperature T^* (see main text's Eq. (1)). Hexagonal symbols correspond to the crystalline phase, and triangles to melting systems, according to the Lindemann criterion. Each point is colored according to the active diffusion coefficient D_A.

- **Supplementary Figure 3**

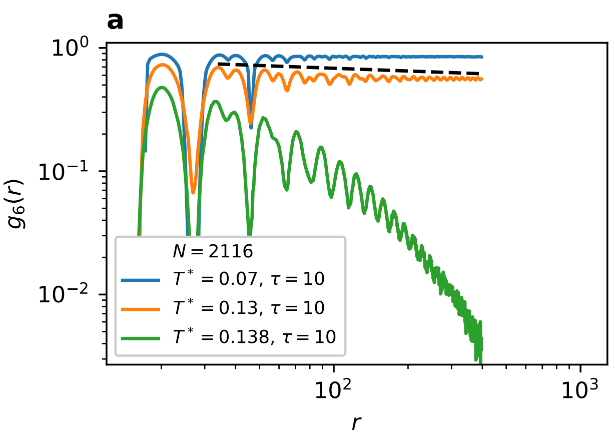

FIG. 3. **Simulations of large and small active systems during melting at large persistence times τ=10.** (a) Correlation function of the hexatic order parameter $g_6(r)$ at various values of the reduced temperature T^* (colored curves, see legend), for a small system size ($N \approx 2000$), and long $\tau = 10$. The dashed line is a fit of the maxima of the orange curve with a power-law to highlight its characteristic decay. The three phases are distinguished by the decay of g_6: solid (blue) not decaying, hexatic (orange) power-law decay, liquid (green) exponential. The same two-step scenario is also shown in (b) for a significantly larger system ($N \approx 16000$) at long $\tau = 10$.

- **Supplementary Figure 4**

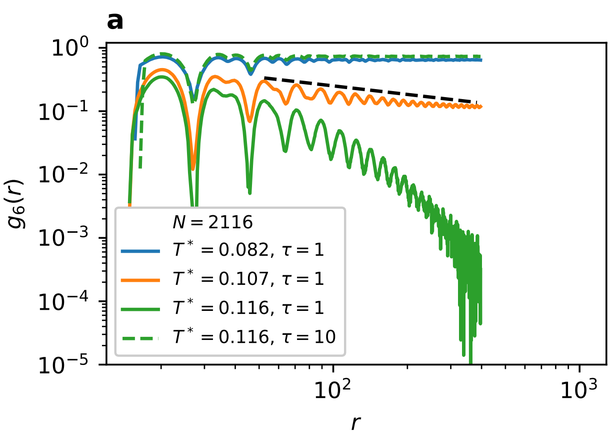

FIG. 4. **Simulations of large and small active systems during melting at different persistence times τ.** (a) Correlation function of the hexatic order parameter $g_6(r)$ at various values of the reduced temperatue T^* (colored curves, see legend), for a small system size ($N \approx 2000$), for $\tau = 1$. The dashed line is a fit of the maxima of the orange curve with a power-law to highlight its characteristic decay in hexatic phase. Comparing the solid and dashed green curves we see that when $\tau = 1$ the system is in the liquid state at $T^* = 0.116$ while, when τ is very long ($\tau = 10$), the system is in the solid phase at the same T^*. This signals that system melts at higher T^* at longer τ-values. The same situation is also found for a significantly larger system ($N \approx 16000$) as shown in (b).

- **Supplementary Figure 5**

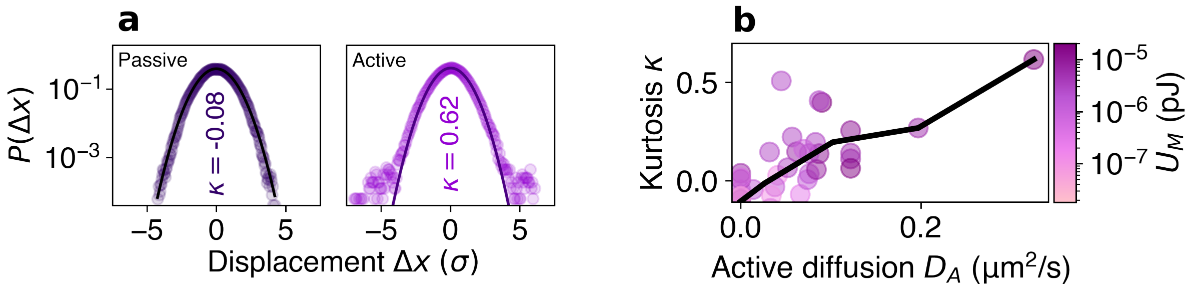

FIG. 5. **Particle kurtosis in the crystal caused by bacterial activity.** (a) Probability distribution of the displacement from the equilibrium (mean) particle position Δx, normalised with each particle's fluctuation amplitude σ_i and fit with a Gaussian function. For the passive case, $U_M = 10^{-7}$ pJ $D_A = 0$, $\sigma_i = 1.02\mu m$. For the active case, $U_M = 10^{-5}$ pJ $D_A = 0.3\mu m^2/s$, $\sigma_i = 0.97\mu m$. (b) Kurtosis of $P(\Delta x)$ as a function of D_A for all U_M in the crystalline phase and averaged curve.

- **Supplementary Figure 6**

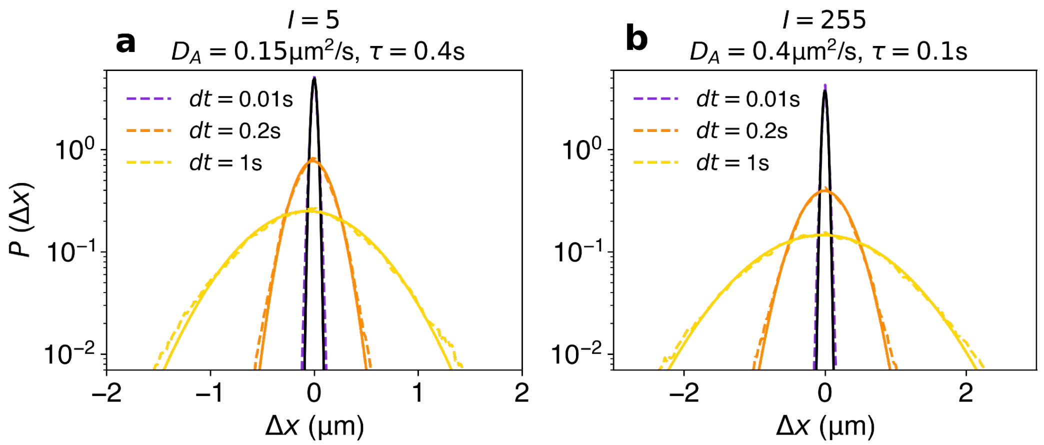

FIG. 6. **Particle displacements in absence of external forces.** Probability distribution of colloids the displacements at different times (see legend) and activity levels (see figures title). Measurements correspond to free particles (no magnetic field, $H = 0$). The continuous lines are fits with Gaussian functions. (a) At low light intensity. (b) At high light intensity.

* helena.massanacid@uniroma1.it
† claudio.maggi@cnr.it
