# Supplementary material for: Multiple temperatures and melting of a colloidal active crystal
Source: Nat Commun. 2024 Aug 3;15:6574. doi: 10.1038/s41467-024-50937-2 (PMC11297967; doi:10.1038/s41467-024-50937-2)
Supplement: Supplementary file 3 — Description of Additional Supplementary Files [file 41467_2024_50937_MOESM3_ESM.pdf]

# Description of Additional Supplementary Files of Multiple temperatures and melting of a colloidal active crystal

Helena Massana-Cid,[1,*] Claudio Maggi,[2,1,†] Nicoletta Gnan,[3,1] Giacomo Frangipane,[1,2] and Roberto Di Leonardo[1,2]

[1]*Dipartimento di Fisica, Sapienza Università di Roma, Piazzale A. Moro 5, 00185 Rome, Italy*
[2]*NANOTEC-CNR, Soft and Living Matter Laboratory, Institute of Nanotechnology, Piazzale A. Moro 5, 00185 Rome, Italy*
[3]*CNR Institute of Complex Systems, Uos Sapienza, Piazzale A. Moro 5, 00185 Rome, Italy*

## Supplementary Movies

- File Name: Supplementary Movie 1

  Description: This video illustrates the assembly of paramagnetic colloids in a two-dimensional colloidal crystal via a constant and perpendicular magnetic field with amplitude $H = 10$mT in a bath of swimming photokinetic bacteria. The video corresponds to Fig.1b of the article.

- File Name: Supplementary Movie 2

  Description: This video illustrates side by side the active (green light is ON, bacteria are moving and pushing the colloids) and passive (light is OFF, bacteria do not move) configurations of the same crystal. The magnitude of the magnetic field with is $H = 10$mT. The video corresponds to Fig.1c of the article.

- File Name: Supplementary Movie 3

  Description: This video illustrates passive melting in the absence of activity and bacteria (constant $D_A$=0), controlled by the magnetic field $H$. It shows on the top a crystalline system (Moderate $U_M = 1{\cdot}10^{-7}$pJ), and a melting crystal on the bottom (low $U_M = 2{\cdot}10^{-8}$pJ). The video corresponds to part of Fig.3a of the article.

- File Name: Supplementary Movie 4

  Description: This video illustrates active melting, controlled by bacterial speed and tuned by light. It shows on the top a crystalline system (No activity, i.e. $D_A$=0, light is off), and a melting crystal on the bottom ($D_A = 0.1\mu$m$^2$/s, light is ON). The magnitude of the magnetic interaction energy is constant $U_M = 2 \cdot 10^{-7}$pJ. The video corresponds to part of Fig.3a of the article.

* helena.massanacid@uniroma1.it
† claudio.maggi@cnr.it
